# Quantum Digital Signature Using Entangled States for Network

**DOI:** 10.3390/e27111179

**Published:** 2025-11-20

**Authors:** Changho Hong, Youn-Chang Jeong, Osung Kwon, Se-Wan Ji

**Affiliations:** The Affiliated Institute of ETRI, Yuseong-daero 1559, Yuseong-gu, Daejeon 34044, Republic of Korea

**Keywords:** quantum communication, quantum network, quantum digital signature, one-time signature

## Abstract

We propose an entanglement-based quantum digital signature (QDS) protocol optimized for quantum networks. The protocol follows the Lamport-inspired QDS paradigm but eliminates QKD post-processing by signing and verifying with raw conclusive keys, thereby reducing latency and implementation complexity. We provide a finite-size security analysis of robustness, unforgeability, and non-repudiation. Under standard fiber-loss and detector models, simulations show a consistent signature rate advantage over a representative Lamport-inspired QDS baseline across metro-to-regional distances. The proposed protocol is practical for near-term deployment while preserving end-to-end, finite key security guarantees.

## 1. Introduction

Digital signature (DS) is a cryptographic service that ensures that an important message was created by the legitimate and clearly identified sender (authenticity), that the message was not tampered with (integrity), and that the message transmitter cannot deny having sent the message (non-repudiation). Quantum digital signature (QDS) should also provide these services. The most common DS uses a public key system consisting of a private key and a public key [1]. The signer chooses a message and uses private key to generate a signature $sig\left(m\right)$. A verifier of signatures checks whether to accept the message as originating from the legitimate signer or not by using the public key. The security of these schemes is based on the assumption that attackers have limited computational power and that solving mathematical problems such as discrete logarithms or factorization is hard for them. In other words, if an extremely powerful computer is built, it could solve these problems efficiently and break the security of these DS schemes. In 2001, Gottesman and Chuang proposed the first QDS scheme [2]. The protocol uses quantum one-way function, where the inability to invert is not based on computational assumption but assured by the law of quantum mechanics. The quantum one-way function exploits the fact that non-orthogonal quantum states cannot be distinguished perfectly. If we have a quantum state $\left|\left.g(x)\right\rangle \right.$, where g(x) represents the classical described state, and the set of possible states are non-orthogonal, nobody should be able to settle the classical description of the state with high probability.

Inspired by Lamport’s one-time signature (OTS) scheme [3], which relies solely on the one-wayness of hash functions for security, QDS protocols adopt the same principle of partial key revelation per message while leveraging quantum key distribution (QKD) to provide information theoretically secure key establishment [2,4]. This hybridization eliminates the need for large classical public keys, enhances forward security, and ensures robustness even against quantum adversaries [5]. Recent advances have produced practical instantiations, notably one-time universal hashing (OTUH) QDS [5], which compresses arbitrary length messages into fixed-size digests and uses QKD-generated keys without privacy amplification, thereby enabling efficient, low-latency signing [5]. Furthermore, measurement device independent (MDI) QDS protocols [6] have removed detector side channel vulnerabilities, while chip-integrated implementations [7] have demonstrated scalable, low-cost deployment over metropolitan-scale fiber networks [7,8,9]. Experimental work has further pushed these concepts into the field: Yin et al. demonstrated an MDI-QDS protocol over a metropolitan network with security level on the order of 10^−7^, securing binary messages against all detector side channel attacks [10]; Liu et al. achieved decoy-state QDS over 102 km of fiber, even signing a 32 bit message (“USTC”) at 51 km [11]; Clarke et al. additionally showed equivalent QDS performance over ≈134 km (≈43 dB loss) in installed fiber, setting records for transmission distance [7,12,13,14]. Together, these developments position Lamport-inspired QDS (QS-L) as a promising candidate for securing critical communications in quantum era infrastructure.

In this paper, we propose a QDS protocol based on entangled states. This protocol belongs to the QS-L family of protocols and share several common features. QS-L integrates QKD without post-processing (i.e., omitting full error correction and privacy amplification) and parameters that are standard in QKD are used as pass/fail criteria in the QDS verification phase. The raw key, which is the intermediate data or key of QKD, is used as the signature key. It means that QS-L family has significant advantages in implementation [8,15]. Although quantum secure direct communication (QSDC) may, in the ideal case, dispense with post-processing, the manner in which post-processing is omitted in QSDC differs from what we mean by eliminating post-processing in the proposed QDS. QSDC transmits plaintext directly through quantum states and thereby dispenses with key distillation, typically employing block coding and error correction, and in some variants, device independence techniques [16,17,18,19,20]. In our protocol we do not transmit the message in quantum states and we use the quantum channel only to generate correlated classical strings that are later tested for signature verification, and by “no QKD post-processing”, we mean that full error correction and privacy amplification are not applied while decoy-state estimation and thresholding are still performed.

There are some assumptions on the QS-L family as follows.

- Alice–Bob and Alice–Charlie are connected by imperfect quantum channels.
- Alice–Bob and Alice–Charlie are connected by authenticated classical channels.
- Bob–Charlie link is confidential and authenticated, and its content (indices, test positions) is never revealed to Alice. (In our symmetrization-free QDS this assumption is unnecessary, and the reasons will become clear as the protocol is presented.)
- In the key generation step within the distribution phase, participating users all act honestly.

## 2. Quantum Digital Signature Using Entangled States

The protocol suggested in this paper consists of a distribution phase, estimation phase, and a messaging phase. The distribution phase comprises two steps: key generation and reordering. In the estimation phase, we derive the thresholds necessary to validate the signature. The message phase consists of signature and verification. In the proposed QDS protocol, Alice is the signer, Bob the authenticator, and Charlie the verifier [21].

Our protocol uses the SARG04 [22] encoding/decoding method; a brief description follows. Because our scheme operates with entangled states, the single-photon formulation of the original SARG04 is modified as follows. In the proposed entanglement-based SARG04 protocol, polarization-entangled photon pairs are distributed to Alice and Bob (Charlie), who each measure every qubit in a randomly chosen x-basis or z-basis and record the outcomes. During sifting, Alice first publicly announces, for every round, a pair of non-orthogonal candidate states that contains the state consistent with her setting and outcome, chosen from $\left[\left\{\left|\left.0\right\rangle \right.,\left|\left.+\right\rangle \right.\right\},\;\left\{\left|\left.+\right\rangle \right.,\left|\left.1\right\rangle \right.\right\},\left\{\left|\left.1\right\rangle \right.,\left|\left.-\right\rangle \right.\right\},\left\{\left|\left.-\right\rangle \right.,\left|\left.0\right\rangle \right.\right\}\right]$. Given this announcement, Bob (Charlie) classifies the round as conclusive if and only if his projective measurement outcome is orthogonal to exactly one of the two candidates; in that case, the other candidate is inferred as the emitted state and the raw bit is set by its basis label (z→0, x→1). Otherwise, the round is discarded. No prior disclosure of Bob’s basis is required for decoding (basis information is revealed only for parameter estimation samples). The surviving conclusive events are then used for estimation of error. For brevity, we designate the above procedure as the bit assignment process (BAP).

### 2.1. The Distribution Phase

The distribution phase can be explained by dividing into the key generation step and the reordering step. Among them, the key generation step corresponds to the post-processing free QKD process.

The purpose of the distribution phase is to create keys for signing and verifying, and to securely share them between legitimate users, thereby providing a non-repudiation service.

#### 2.1.1. Key Generation Step

Alice randomly generates an entangled state of

(1) $${\left|\left.\psi^{z}\right\rangle \right.}_{AB}=\frac{1}{\sqrt{2}}\left({\left|\left.00\right\rangle \right.}_{AB}+{\left|\left.11\right\rangle \right.}_{AB}\right)$$

(2) $${\left|\left.\psi^{x}\right\rangle \right.}_{AB}=\frac{1}{\sqrt{2}}\left({\left|\left.++\right\rangle \right.}_{AB}+{\left|\left.--\right\rangle \right.}_{AB}\right)$$

and shares it with Bob. Similarly, Alice creates an entangled state of

(3) $${\left|\left.\psi^{z}\right\rangle \right.}_{AC}=\frac{1}{\sqrt{2}}\left({\left|\left.00\right\rangle \right.}_{AC}+{\left|\left.11\right\rangle \right.}_{AC}\right)$$

(4) $${\left|\left.\psi^{x}\right\rangle \right.}_{AC}=\frac{1}{\sqrt{2}}\left({\left|\left.++\right\rangle \right.}_{AC}+{\left|\left.--\right\rangle \right.}_{AC}\right)$$

and shares it with Charlie. Here, the subscripts AB and AC mean that the two-qubit entangled state is shared between Alice and Bob, and Alice and Charlie, respectively. The states $\left|\left.\psi^{z}\right\rangle \right.$ and $\left|\left.\psi^{x}\right\rangle \right.$ represent the z-basis entangled state and the x-basis entangled state, respectively. The rules for Alice’s preparation and distribution of quantum states are as follows. For each possible message $m=\left\{0,1\right\}$, Alice prepares two types of entangled quantum state sequences with length n—that is sequence ${\left(A_{b}\right)}_{m}$, ${\left(B_{a}\right)}_{m}$, ${\left(A_{c}\right)}_{m}$ and ${\left(C_{a}\right)}_{m}$. The sequence ${\left(A_{b}\right)}_{m}$ and ${\left(B_{a}\right)}_{m}$ form n entangled states, and similarly, the sequence ${\left(A_{c}\right)}_{m}$ and ${\left(C_{a}\right)}_{m}$ also form n entangled states. Here, each entangled state constituting sequence is randomly selected and generated from $\left\{\left|\left.\psi^{z}\right\rangle \right.,\left|\left.\psi^{x}\right\rangle \right.\right\}$. Each sequence of length N includes the following decoy states. The decoy method is commonly used in QKD implementation for secure communication [23,24,25]. It is a powerful tool that can be used to improve the security and performance of QKD. The decoy method is also used to set the signature verification threshold in our signature scheme. In the decoy method, Alice uses intensity μ to generate the entangled states used for the signal states, and uses intensity ν and 0 (vacuum) for parameter estimation. When using intensity ν, it is not necessary to generate entangled states, but one of $\left\{\left|\left.0\right\rangle \right.,\left|\left.1\right\rangle \right.,\left|\left.+\right\rangle \right.,\left|\left.-\right\rangle \right.\right\}$ is randomly selected to create a pair of identical states. For example, a pair of $\left|\left.1\right\rangle \right.$ state is generated, and one qubit is stored by Alice herself and the other qubit is transmitted to the opponent. The intensities μ, ν, and 0 are generated with probabilities $p_{\mu }$, $p_{\nu }$, and $p_{0}$, respectively. Here, the state generated with intensity μ is an entangled state, and the state generated with ν is one of $\left\{\left|\left.0\right\rangle \right.,\left|\left.1\right\rangle \right.,\left|\left.+\right\rangle \right.,\left|\left.-\right\rangle \right.\right\}$.

1. Alice sends ${\left(B_{a}\right)}_{m}$ to Bob and ${\left(C_{a}\right)}_{m}$ to Charlie. She measures her states on a randomly chosen basis, thereby forming a sequence of outcomes ($\left({AB}_{a}\right)$ and $\left({AC}_{a}\right)$).
2. Bob and Charlie make a measurement on each state of received sequence (${\left(B_{a}\right)}_{m}$ and ${\left(C_{a}\right)}_{m}$, respectively) by randomly selecting from the measurement bases, z- or x-basis. They announce all the click (measured) events through an authenticated channel to Alice. Only part of quantum states can be detected due to channel loss and imperfect detection [26,27,28,29,30,31]. Alice and Bob throw away all the events that have not clicked on either side. It means that they keep the left data of length *l* (l<n), denoted as ${\left(Ab\right)}_{m}$, kept by Alice and ${\left(Ba\right)}_{m}$ kept by Bob. Alice and Charlie also repeat the same process to form ${\left(Ac\right)}_{m}$ kept by Alice and ${\left(Ca\right)}_{m}$ kept by Charlie. Of course, there is no correlation between the states of sequences of Bob and Charlie at this stage because Alice randomly and independently generated and distributed entangled states.
3. Alice announces the intensity information of all qubits. According to the intensity data, the legitimate three users divide each of their sequence into three strings. For example, Bob divides ${\left(Ba\right)}_{m}$ into ${\left(Ba\right)}_{m}^{\mu }$, ${\left(Ba\right)}_{m}^{\nu }$, and ${\left(Ba\right)}_{m}^{o}$. In a similar manner, Charlie partitions the ${\left(Ca\right)}_{m}$ sequence. We describe these three intensities as λ ($\in \left\{\mu ,\nu ,0\right\}$.

#### 2.1.2. Reordering Step

1. Alice, using the sequence ${\left(Ab\right)}_{m}^{\lambda }$ as a reference, reorders the positions of each bit within the sequence ${\left(Ac\right)}_{m}^{\lambda }$ to match the bit sequence of the sequence ${\left(Ab\right)}_{m}^{\lambda }$. This rearranged sequence is denoted as ${\left({\left(Ac\right)}_{m}^{\lambda }\right)}^{\prime }$. Alice shares the repositioning information with Charlie, allowing Charlie to transform the sequence ${\left(Ca\right)}_{m}^{\lambda }$ to the sequence ${\left({\left(Ca\right)}_{m}^{\lambda }\right)}^{\prime }$ accordingly. We use parentheses and prime (′) to indicate that a sequence has been reordered. This procedure effectively eliminates the canonical symmetrization step in standard QS-L [21].

At this stage, the ownership status of each sequence and the inter-sequence relationships are compiled in Table 1. As a result of the reordering step, ${\left(Ab\right)}_{m}^{\lambda }$ and ${\left({\left(Ac\right)}_{m}^{\lambda }\right)}^{\prime }$ consist of the same bit sequence.

2. They generate new bit strings through the BAP; specifically, Alice obtains ${\left(kA\right)}_{m}$, Bob obtains ${\left(kB\right)}_{m}$, and Charlie obtains ${\left(kC\right)}_{m}$. Note that Bob and Charlie do not reveal which bits are conclusive results.

### 2.2. Estimation Phase

At this phase, three legitimate users determine the authentication security threshold and the verification security threshold of the signature based on the bit error rate. The detailed process is as follows.

1. The signer Alice chooses the authenticator from among Bob and Charlie; the remaining user naturally becomes the verifier of the signature. In this protocol, the authenticator serves as a kind of intermediary for non-repudiation services. The verifier plays the role of validator, verifying Alice’s signature. For the description of the protocol, we suppose that Bob is the authenticator and Charlie is the verifier.
2. Alice, Bob, and Charlie publicly announce all data about ν-, and 0-sequences: Alice’s candidate pair announcements and the measurement outcomes of Bob and Charlie.
3. They estimate the bit error rate of entangled pairs in μ sequences (between ${\left(kA\right)}_{m}^{\mu }$ and ${\left(kB\right)}_{m}^{\mu }$, and ${\left(kA\right)}_{m}^{\mu }$ and ${\left(kC\right)}_{m}^{\mu }$) using the all data of ν sequence (between ${\left(kA\right)}_{m}^{\nu }$ and ${\left(kB\right)}_{m}^{\nu }$, and ${\left(kA\right)}_{m}^{\nu }$ and ${\left(kC\right)}_{m}^{\nu }$) and 0 sequence (between ${\left(kA\right)}_{m}^{0}$ and ${\left(kB\right)}_{m}^{0}$, and ${\left(kA\right)}_{m}^{0}$ and ${\left(kC\right)}_{m}^{0}$). In other words, the bit error rate for the central sequence (*μ* sequence) is calculated by the decoy method commonly used in QKD.
4. Charlie randomly selects a proportion of u in the μ sequence ${\left(kC\right)}_{m}^{\mu }$ to use as test bits, then requests Alice to announce the bit values at those locations. We describe test bit sequences as ${\left({\left(kA\right)}_{m}^{\mu }\right)}_{T}$, ${\left({\left(kB\right)}_{m}^{\mu }\right)}_{T}$, and ${\left({\left(kC\right)}_{m}^{\mu }\right)}_{T}$. Also, let us denote $e_{ab,T}$ and $e_{ac,T}$ as the mismatch rate of conclusive results between ${\left({\left(kA\right)}_{m}^{\mu }\right)}_{T}$ and ${\left({\left(kB\right)}_{m}^{\mu }\right)}_{T}$, and ${\left({\left(kA\right)}_{m}^{\mu }\right)}_{T}$ and ${\left({\left(kC\right)}_{m}^{\mu }\right)}_{T}$, respectively. In QKD, just as in the method used to determine post-processing based on the bit error rate, if $e_{ab,T}$ and $e_{ac,T}$ are too high, the subsequent steps are not performed.
5. Bob and Charlie estimate the conclusive event rates on ${\left(kB\right)}_{m}$ and ${\left(kC\right)}_{m}$. These are denoted as $P_{b}$ and $P_{c}$, respectively. Under ideal statistics, $P_{b}$ and $P_{c}$ are expected to approach 1/4. If they deviate substantially from this nominal value, the protocol is aborted.
6. Based on $e_{ab,T}$, $e_{ac,T}$, $P_{b}$, and $P_{c}$, Alice, Bob, and Charlie set the authentication security threshold $T_{a}$ and verification security thresholds $T_{v}$. Set $T_{a}=e_{ab,T}+\Delta_{A},\;T_{v}=e_{ac,T}-\;\Delta_{V}$ where $\Delta_{A},\;\Delta_{V}>0$ are one-sided finite-size margins derived from the conclusive sample sizes [32,33]. The thresholds are required to satisfy $0<T_{a}<T_{v}<1/2$. In other words, by excluding the post-processing procedures that are essential, in general, QKD when setting up $T_{a}$ and $T_{v}$, the comprehensive effects of losses and errors included in the shared key are reflected.
7. The three legitimate users discard the test bits and keep the remaining bits in μ strings with length $L_{k}$. We denote these remaining bit sequences as ${\left(K_{a}\right)}_{m}$, ${\left(K_{b}\right)}_{m}$, and ${\left(K_{c}\right)}_{m}$.

### 2.3. Message Phase

1. Alice sends the message and the corresponding signature $\left\{m,\;{\left(K_{a}\right)}_{m}\right\}$ to the authenticator Bob to sign message m.
2. Bob receives $\left\{m,\;{\left(K_{a}\right)}_{m}\right\}$ and estimates the error rate $e_{ab}^{\prime }$ between ${\left(K_{a}\right)}_{m}$ and ${\left(K_{b}\right)}_{m}$. If $e_{ab}^{\prime }<\;T_{a}$, Bob accepts the signature and transmits $\left\{m,\;{\left(K_{a}\right)}_{m}\right\}$ to the verifier Charlie; otherwise, he aborts the signature and announces the failure result.
3. In a similar way to Bob, Charlie calculates the error rate $e_{ac}^{\prime }$ between ${\left(K_{a}\right)}_{m}$ and ${\left(K_{c}\right)}_{m}$. Charlie accepts the signature if $e_{ac}^{\prime }<T_{v}$. As a result, Charlie accepts $\left\{m,\;{\left(K_{a}\right)}_{m}\right\}$ as Alice’s signature for message m with signature verification using $T_{a}$ and $T_{v}$.

## 3. Security Analysis

This section provides a comprehensive and rigorous security analysis of the proposed QDS protocol utilizing entangled states. We address robustness, unforgeability, and non-repudiation by systematically applying methods from the established literature [21]. Taken together, these results establish that the signature scheme achieves information theoretic security while supporting efficient message authentication. Here, we discuss the security of the proposed protocol from these three perspectives.

### 3.1. Robustness

Robustness is defined as the probability of honest participants successfully completing the protocol without aborting due to unexpected discrepancies. Although robustness pertains to honest run completeness rather than adversarial security, it is standard to report it alongside security parameters. Thus, including robustness in the security section is appropriate. In this protocol, robustness is achieved by setting an error rate threshold that distinguishes acceptable noise (due to channel imperfections) from adversarial interference (indicative of an attack). We compute the probability $\epsilon_{rob}$ that the protocol fails because of noise or errors by using Chernoff bound [10]. The Chernoff bound yields a tail probability for deviations in the empirical error from its expectation. Through the estimation step of our QDS, legitimate users calculate the error rate between transmitted and received qubit sequences. By leveraging random sampling without replacement, the probability of an honest abort, $\epsilon_{rob}$, can be defined as

(5) $$\epsilon_{rob}=exp\left[-2n^{cu}{\left(T_{a}-E_{B}^{cu}\right)}^{2}\right],\;\mathrm{for}\;E_{B}^{cu}<T_{a}$$

where $T_{a}$ is the acceptance authentication threshold, $E_{B}^{cu}$ is the measured bit error rate between Alice and Bob for the conclusive bits, and $n^{cu}$ is the length of the conclusive bit sequence used for authentication. From a robustness perspective, Equation (5) indicates that the probability of protocol abortion exponentially decreases as the difference between the measured bit error rate $E_{B}^{cu}$ and the threshold $T_{a}$ increases. Hence, to ensure high robustness, the threshold $T_{a}$ should be appropriately selected, considering the expected system imperfections and channel conditions.

### 3.2. Unforgeability

The protocol’s unforgeability is maintained by ensuring that an adversarial party (e.g., an internal forger such as Bob) cannot replicate or manipulate Alice’s signature to deceive other participants. The forgery probability $\epsilon_{for}$ quantifies the chance that an adversary (e.g., authenticator Bob) successfully forges the signature to deceive the verifier Charlie. Let us explain unforgeability based on our QDS protocol.

(1) Estimate the lower bound of the secure single-photon pair events $s_{11}^{AC}$ between Alice and Charlie by decoy-state analysis:
  (6) $$s_{11}^{AC*}\geq \frac{p_{\mu }^{2}e^{2\mu }}{\upsilon^{2}{\left(\mu -\nu \right)}^{2}n_{p}^{AC}}{\left(\mu^{2}e^{\nu }\frac{n_{\nu }^{AC}}{p_{\nu }}-\mu^{2}e^{\mu }\frac{n_{\mu }^{AC}}{p_{\mu }}+\left(\nu^{2}-\mu^{2}\right)\frac{n_{0}^{AC}}{p_{0}}\right)}^{2}$$
  The meaning of each variable is specified below.
  - $n_{p}^{AC}$: Observed number of conclusive detection events on the Alice–Charlie link when the source intensity is $p\in \left\{\mu ,\nu ,0\right\}$.
  - $n_{\mu }^{AC}$, $n_{\nu }^{AC}$, $n_{0}^{AC}$: Shorthand for the above counts at the signal (μ), weak decoy (ν), and vacuum (0) settings, respectively.
  - $e^{2\mu }$, $e^{2\nu }$: Poisson weight factors appearing in the decoy linear relations for pair sources when isolating the single-photon contribution from $n_{p}^{AC}$.
(2) Compute the maximum number of single-photon error events $t_{11}^{AC}$:
  (7) $$t_{11}^{AC*}\leq \frac{p_{\mu }^{2}\mu^{2}e^{2\mu }}{\upsilon^{2}n_{p}^{AC}}\left(e^{\nu }\frac{n_{\nu }^{AC}}{p_{\nu }}-\frac{n_{0}^{AC}}{2p_{0}}\right)\left(e^{\nu }\frac{n_{\nu }^{AC}}{p_{\nu }}-\frac{n_{0}^{AC}}{p_{0}}\right)$$
(3) Calculate minimum expected mismatch rate $E_{BF11}^{*}$:
  (8) $$E_{BF11}^{*}=\frac{t_{11}^{AC*}}{s_{11}^{AC*}}$$
(4) Bound the forgery success probability using Chernoff bound [10]:

(9) $$\epsilon_{for}=\exp\left[-\frac{{\left(E_{BF11}^{*}-T_{\nu 11}\right)}^{2}}{2E_{BF11}^{*}}s_{11}^{AC*}\right]$$

Here, $T_{\nu \;11}$ represents the verification threshold specifically for single-photon entangled pairs.

From the perspective of forgery attacks, Equation (9) explicitly shows that the probability of a successful forgery by the adversary decreases exponentially with the number of secure single-photon events $s_{11}^{AC*}$ and the squared difference between the expected mismatch rate and the verification threshold. Thus, our QDS protocol ensures strong security against forgery attacks as long as proper thresholds are established and verified. The threshold effectively limits an adversary’s chances of successful forgery.

### 3.3. Repudiation Resistance

Repudiation resistance ensures that Alice cannot deny having signed a message once it is authenticated by Bob and verified by Charlie. To rigorously quantify the security against repudiation, we evaluate the probability $\epsilon_{rep}$ that Alice successfully repudiates her signature. This probability is carefully bounded by statistical methods involving relative Hamming distances and threshold parameters, as explained below in detail.

First, we compute the relative Hamming distance between the bit sequences held by the authenticator Bob and verifier Charlie after the reordering step:

(10) $$\Delta_{BC}^{cu}=\frac{\sum_{i=1}^{n^{cu}}\left|B_{i}-C_{i}\right|}{n^{cu}}$$

Here, $B_{i}$ and $C_{i}$ denote the ith bit in Bob’s and Charlie’s conclusive bit sequence, respectively. $n^{cu}$ is the total length of the conclusive bit sequences shared between Bob and Charlie after discarding inconclusive and test bits. This relative Hamming distance ($\Delta_{BC}^{cu}$) physically represents the proportion of differing bits between Bob’s and Charlie’s conclusive sequences, reflecting discrepancies arising primarily from quantum channel noise and potential adversarial actions.

Next, we solve the following transcendental equation to determine the critical parameter A:

(11) $$\frac{{\left[P_{C}^{c}T_{\nu }-P_{C}^{c}\left(\frac{\Delta_{BC}^{cu}}{n^{cu}}+\frac{A}{P_{B}^{c}}\right)\right]}^{2}}{3P_{C}^{c}\left(\frac{\Delta_{BC}^{cu}}{n^{cu}}+\frac{A}{P_{B}^{c}}\right)}=\frac{{\left(A-P_{B}^{c}T_{a}\right)}^{2}}{2A}$$

The parameters in the above equation are explicitly defined as follows. $P_{B}^{c}$ is the proportion of conclusive measurement results in Bob’s bit sequence, reflecting the ratio of bits for which Bob obtained unambiguous measurement outcomes. $P_{C}^{c}$ is the proportion of conclusive measurement results in Charlie’s bit sequence, analogously defined for Charlie. $\Delta_{BC}^{cu}$ is the relative hamming distance between Bob’s and Charlie’s conclusive bit strings after the reordering step. $T_{\nu }$ is the verification threshold used in the repudiation analysis, i.e., the admissible mismatch limit for Charlie’s acceptance test in the messaging phase. $T_{a}$ is the authentication threshold used by Bob. It is fixed in the estimation phase from conclusive test statistics. A is an auxiliary parameter representing the critical error limit necessary to balance and bound Alice’s potential repudiation probability effectively. Equation (11) essentially quantifies the boundary conditions under which the legitimate users (Bob and Charlie) can confidently reject Alice’s repudiation attempt.

Finally, with A fixed, the repudiation probability can be evaluated as follows:
  (12) $$\epsilon_{rep}=exp\left(-\frac{{\left(A-P_{B}^{c}T_{a}\right)}^{2}}{2A}n^{u}\right)$$

Here, the parameter $n^{u}$ denotes the length of the bit sequence retained after removing test bits, effectively representing the available sample size for verification. Equation (12) indicates that Alice’s probability of successfully repudiating her signature is exponentially suppressed by two factors. The first factor is that a greater difference between the parameter A and the product $P_{B}^{c}{\;T}_{a}$ strengthens security by making repudiation significantly more improbable. The second factor is that increasing the number of retained verification bits ($n^{u}$) drastically reduces the likelihood of successful repudiation, thereby strongly ensuring protocol security. Thus, the derived equation clearly emphasizes the protocol’s ability to securely prevent repudiation, provided thresholds are properly selected and a sufficiently large verification dataset is utilized.

### 3.4. Overall Security Discussion

The overall security of the protocol against forging, repudiation, and robustness failures can thus be given by

(13) $$\epsilon_{tot}=\epsilon_{rob}+\epsilon_{for}+\epsilon_{rep}+\epsilon_{stat}$$

Here, $\epsilon_{stat}$ encompasses additional statistical variations from finite-size effects and experimental inaccuracies.

### 3.5. Conclusion of Security Analysis

Our finite-size security analysis demonstrates end-to-end protection of the proposed QDS against the three standard failure modes—honest aborts, forgery, and repudiation—using statistics obtained in the estimation phase and applied during the message phase.

The honest abort event is controlled by a Chernoff-type tail bound whose tightness improves as the authentication sample size increases and as the observed conclusive bit error remains further below the authentication threshold, yielding rapidly improving robustness under realistic margins. For unforgeability, a decoy-state procedure isolates the single-photon component on the Alice–Charlie link; by lower bounding secure single-photon events, upper bounding single-photon errors, and forming the corresponding minimum expected mismatch, comparison with a single-photon verification threshold yields an exponentially small forgery probability that decreases with both the effective single-photon sample size and the squared gap to that threshold. Repudiation is addressed after the reordering step by measuring the relative Hamming distance between Bob’s and Charlie’s conclusive strings and solving a transcendental relation that combines their conclusive rates with the authentication and verification thresholds to obtain a critical parameter; the resulting repudiation bound decays exponentially with the verification sample size and strengthens as this parameter separates from the acceptance boundary. Finally, the overall failure probability is the sum of the three contributions above together with a residual finite-size term that captures additional statistical and experimental effects, enabling explicit budgeting of security parameters for implementation.

## 4. Realization Discussion

**Figure 1:**
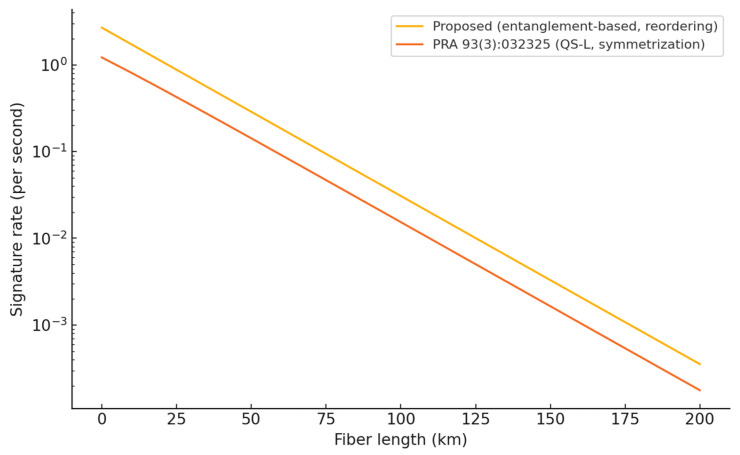
Comparison of our QDS protocol and general QS-L protocol [34].

The proposed QDS protocol was deliberately designed for experimental feasibility and practical deployment. To assess the feasibility under realistic conditions, we compare our protocol with a canonical QS-L scheme implemented over insecure channels [34] using a common channel detector model and finite-size security budgets. The resulting distance-dependent signature rates are plotted in Figure 1, where two curves are shown (the proposed protocol and the QS-L baseline) in accordance with our intended comparison. In brief, the proposed design maintains a consistent rate advantage over the QS-L baseline across the metro-to-regional distance range because it (i) eliminates the data-discard cost of the symmetrization step and (ii) retains conclusive event statistics comparable to prepare-and-measure schemes under the same channel and detector parameters.

**Figure A2:**
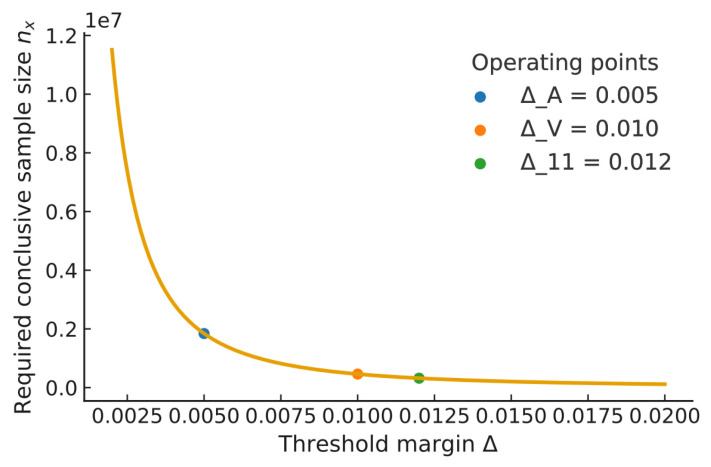
$n_{x}$ versus Δ under $\epsilon_{x}=10^{-10}$.

The parameter values used in the simulations are as follows. All parameters are given per time slot unless otherwise noted. Channel and detection: detection efficiency 52%; dark-count probability $1.3\times 10^{-7}$; basis misalignment 0.15%; insertion loss 1.2 dB; fiber attenuation 0.194 dB/km. Security budgets: $\epsilon_{rob},\;\epsilon_{for},\;\epsilon_{rep}\;\leq 10^{-10}$. Clocking and sifting: repetition rate 100 MHz (typical value in contemporary fiber-based systems); SARG04-style conclusive fraction was fixed to 0.25 (nominal expectation used consistently across both curves). Threshold margins (finite-size): authentication margin $\Delta_{A}=0.005$ (absolute), verification margin $\Delta_{V}=0.01$; single-photon mismatch threshold gap $\Delta_{11}=0.012$ was used to instantiate the forging bound. Tail calibration: Chernoff constant is set to 2 with natural-log tail; the required signature length for each failure event is computed as $2\ln\left(\frac{1}{\epsilon_{x}}\right)/\Delta_{x}^{2}$, and the effective length is the maximum of the three. We plot $n_{x}$ versus Δ for the security budget used here and mark the three operating margins; see Appendix A, Figure A2. Source models: for our protocol, entanglement pair generation probability per clock is fixed at 0.5; for the QS-L baseline (prepare and measure with weak coherent pulses), the mean photon number is set to 0.5 (typical operating point). All “typical” values are standard choices for comparative studies; if device-specific calibrations are available, the curves translate accordingly without changing the qualitative separation.

With identical security budgets ($\epsilon_{rob},\;\epsilon_{for},\;\epsilon_{rep}$) and device parameters, the elimination of the symmetrization step nearly doubles the number of usable conclusive events, which directly translates into a higher signature rate at short and intermediate distances; the gap persists at longer distances until channel loss dominates both curves. Because the bounds in Section 3 scale exponentially with the product of the effective sample size and the squared threshold gap, modest improvements in margins (via calibration) or in conclusive fractions (via collection optics) yield disproportionate gains in rate in practice.

Given that our QDS targets quantum network applications, we utilize entangled states [7,13,14]. This raises a natural question of practical feasibility.

## 5. Integration with Quantum Networks and Deployment Considerations

### 5.1. Network Native Rationale

Our protocol is entanglement-centric by construction: the signing and verification criteria are calibrated from decoy-state statistics. This aligns with the vision of quantum internet in which entanglement is the network service and applications consume it via higher-layer protocols [35]. In particular, the ITU-T Y.3800 framework and related Y.38xx series decompose quantum key distribution networks (QKDNs) into the user, key management, control, and transport strata; entanglement distribution (via fiber or free space) is the primitive that these strata orchestrate [36]. Our use of entangled states as the resource therefore maps naturally onto network operations in which entanglement is generated, routed, or swapped across multiple domains.

### 5.2. Topologies and Relay Placement

The protocol supports (i) endpoint-sourced entanglement, where Alice’s source feeds Bob and Charlie directly (as in our baseline), (ii) network-sourced entanglement, where an entanglement server (ES) injects Bell pairs into a metro network (star or mesh) for multi-tenant consumption, and (iii) an MDI-hub realization, where a central untrusted relay performs Bell-state measurements (BSMs). Metro-scale analyses and field concepts using an ES that streams entanglement to users over line of sight, free space, or fiber links have been reported, validating (ii) at city scale [37]. Option (iii) inherits the MDI advantages and is consistent with our symmetrization step, which equalizes recipients’ evidence even under hub-routed traffic.

### 5.3. Interoperability with Control/Management Planes

Our decoy derived authentication and verification threshold functions as physical layer telemetry that can be surfaced to network controllers. ETSI GS QKD 014 [38] defines a REST key delivery API between QKDNs and applications, while ETSI GS QKD 015 [39] standardizes SDN control interfaces for provisioning, monitoring, and policy enforcement in disaggregated networks. Exposing the measured error rates and threshold margins ($e_{obs}-\left\{T_{auth},\;T_{ver}\right\}$) as metrics allows controllers to (a) select routes/relays with adequate quantum signal quality, (b) adjust intensity probabilities ($p_{\mu },\;p_{\nu },p_{0}$) under congestion or weather dynamics, and (c) schedule QDS signing windows when decoy inferred yields are optimal—without changing the security model.

### 5.4. Empirical Evidence from Network Trials

A three-party MDI-QDS field test over a ~200 km^2^ metropolitan network demonstrated successful binary message signing with security level ~$10^{-7}$, removing detector side channels via an untrusted relay—directly supporting our (iii) deployment mode [10]. This complements long-running QKD network trials that have integrated heterogeneous links and centralized key management (e.g., Tokyo QKD Network, SECOQC Vienna), establishing operational practices for multi-vendor, SDN-assisted quantum networks [40,41].

### 5.5. Scalability via Integrated Photonics

Large scale adoption hinges on cost, footprint, and stability of quantum transceivers. Silicon photonic QKD has achieved metropolitan field rates with monolithically integrated encoders and receivers, demonstrating stability and manufacturability compatible with carrier-grade deployment [42]. Recent work reports gigabit class secret key rates on chip at 10 km, while integrated CV-QKD receivers have operated over tens of kilometers, suggesting a path to compact, low-cost entanglement distribution and detection hardware for QDS overlays [43,44]. Although our protocol uses DV entanglement, the same integration ecosystem (PICs, packaging, and classical DSP) applies to entanglement sources, BSM nodes, and time/frequency multiplexed distribution.

### 5.6. Extending Reach with Repeaters and Swapping

For backbone scale deployment, entanglement swapping and quantum repeaters extend feasible distances by composing high-fidelity pairs across segments [45,46]. Our entanglement-based signing naturally composes with repeater chains: the distribution phase operates over swapped Bell pairs, while the decoy analysis and thresholding continue to bound single pair yields and error events end-to-end. This is consistent with recent continental scale QKD network reports (e.g., China’s carrier-grade CN-QCN over 10,000 km with multi-type hybrid networking) and with space to ground entanglement/QKD progress that anticipates global integration [47,48].

### 5.7. Operational Mapping of Protocol Steps

In networks, (a) key generation maps to entanglement provisioning (ES or repeater chain) with decoy scheduling per path; (b) symmetrization becomes a control plane function that publishes index maps (or PRNG seeds) to verifiers over authenticated classical channels; and (c) estimation act as link state measurements, feeding SDN policies for route selection and admission control. The Messaging phase can be executed at the network edge or via an MDI hub: in the latter case, our verifier’s acceptance thresholds are evaluated using keys derived from BSM outcomes, preserving non-repudiation while improving device independence [10].

### 5.8. Summary

Entangled states are not merely a resource used by our scheme—they are the core service envisioned for future quantum networks, where entanglement distribution is orchestrated much like bandwidth provisioning in classical networks [35,36]. Our protocol is therefore “network native”: it consumes entanglement directly as the signing key material, relies on decoy-state statistics that can be interpreted as physical layer quality metrics, and exposes clear acceptance thresholds that network controllers can monitor for routing and scheduling. This alignment allows the protocol to plug seamlessly into standardized interfaces (e.g., ITU-T Y.3800, ETSI GS QKD 014/015) and benefit from network-level optimizations such as path selection, load balancing, and adaptive intensity control. Furthermore, its reliance on raw keys and minimal post-processing makes it latency-efficient and well-suited for time sensitive applications such as distributed ledgers or control signaling. Finally, because entanglement swapping and repeaters extend network reach, the same distribution phase naturally scales to metro to backbone deployments while preserving finite key, decoy-calibrated security guarantees. In this way, the proposed QDS is positioned not as a standalone cryptographic primitive but as an integral quantum network application layer, bridging rigorous information theoretic security with practical, carrier-grade deployment.

## 6. Conclusions

We have presented an entanglement-based QDS that organizes distribution, estimation, and the messaging phase centered on a reordering step that replaces the symmetrization in conventional QS-L variants. The protocol determines authentication and verification thresholds from decoy-state and conclusive event statistics and operates without assuming a secure Bob–Charlie classical link, while preserving the balance of evidence required for signature verification.

Our finite-size analysis provides explicit bounds for robustness, unforgeability, and repudiation. These bounds are governed by the effective sample sizes and by the gaps between observed mismatch rates and the thresholds $T_{a}$ and $T_{v}$ derived from decoy-state statistics, which yield exponential suppression of all three failure probabilities under practical parameter choices. Overall security is followed by composition, with a residual statistical term to account for finite-size effects.

To gauge feasibility, we compared the distance-dependent signature rates of our protocol with a QS-L baseline under a common channel and detector model and the same security budget. Across the metro-to-regional distance range considered in Figure 1, the proposed design maintains a consistent advantage, and the simulation parameters align with the device and channel assumptions stated in the main text. Together with the network-integration options outlined in Section 5, these results indicate readiness for near-term deployment on quantum network testbeds.

Future work. We will implement a laboratory prototype based on the present protocol and validate end-to-end signing under calibrated device settings. Building on this prototype, we plan a field trial on a campus- or metro-scale fiber network to verify signature generation and verification under real-network conditions and to exercise the reordering workflow alongside the network-integration options discussed in Section 5.

## Figures and Tables

**Table 1:** The ownership status of each sequence.

User/Source	Sequence	
Source	${\left|\left.\psi^{z(x)}\right\rangle \right.}_{AB}$	${\left|\left.\psi^{z(x)}\right\rangle \right.}_{AC}$
Alice	${\left(Ab\right)}_{m}^{\lambda }$	${\left({\left(Ac\right)}_{m}^{\lambda }\right)}^{\prime }$ $(={\left(Ab\right)}_{m}^{\lambda }$)
Bob	${\left(Ba\right)}_{m}^{\lambda }$	
Charlie		${\left({\left(Ca\right)}_{m}^{\lambda }\right)}^{\prime }$

**Figure A1:**
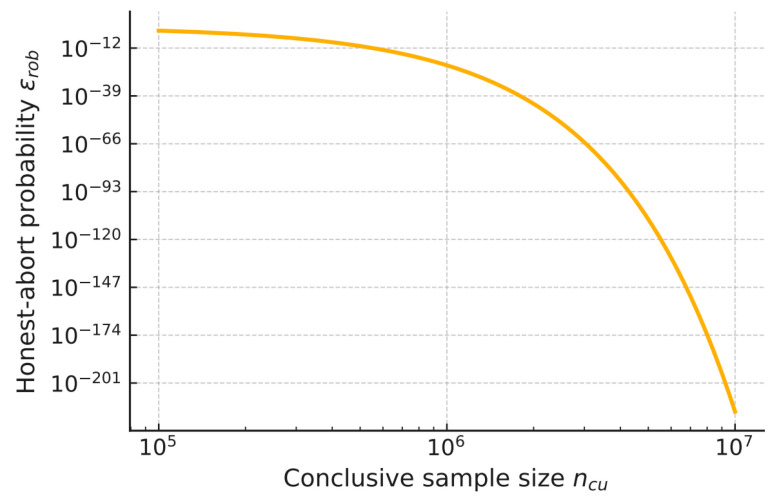
$\epsilon_{rob}$ versus $n_{cu}$ for $\Delta_{A}=0.005$.

## Data Availability

The original contributions presented in this study are included in the article. Further inquiries can be directed to the corresponding author.

## Appendix A. Finite-Size Calibration Examples

We instantiate the finite-size bounds with the parameterization used in this work. First, the robustness bound in Equation (5) reads

$$\epsilon_{rob}=exp\left[-2n^{cu}{\left(T_{a}-E_{B}^{cu}\right)}^{2}\right],$$

so that, for a fixed acceptance margin $\Delta_{A}\equiv T_{a}-E_{B}^{cu}$, the honest-abort probability decays exponentially with the conclusive sample size $n^{cu}$.

Next, adopting the tail-calibration rule used in Section 4, the required conclusive sample size for a target failure budget $\epsilon_{x}$ and margin Δ is as follows:

$$n_{x}=\frac{2\;ln(\frac{1}{\epsilon_{x}})}{\Delta^{2}}$$

For the common budget $\epsilon_{rob}=\epsilon_{for}=\epsilon_{rep}=10^{-10}$ and the margins employed in our simulations (Section 4), the resulting sizes are as follows:

$$\Delta_{A}=0.005\to \;n_{A}\approx 1.842\times 10^{6}$$

$$\Delta_{V}=0.01\to \;n_{V}\approx 4.605\times 10^{5}$$

$$\Delta_{11}=0.012\to \;n_{11}\approx 3.20\times 10^{5}$$

Accordingly, the effective signature length is $n_{eff}=max\left\{n_{A},n_{V},n_{11}\right\}=n_{A}$, which is consistent with our rate–distance evaluation. Figure A1 visualizes $\epsilon_{rob}$ versus $n_{cu}$ for $\Delta_{A}=0.005$, directly exhibiting the exponential suppression predicted by Equation (5). Figure A2 plots $n_{x}$ versus Δ under $\epsilon_{x}=10^{-10}$ and marks the three operating margins used in our study. The 1$/\Delta^{2}$ dependence highlights the benefit of modest calibration improvements to Δ.
